# Association of soluble tumor necrosis factor receptors 1 and 2 with nephropathy, cardiovascular events, and total mortality in type 2 diabetes

**DOI:** 10.1186/s12933-016-0359-8

**Published:** 2016-02-29

**Authors:** Axel C. Carlsson, Carl Johan Östgren, Fredrik H. Nystrom, Toste Länne, Pär Jennersjö, Anders Larsson, Johan Ärnlöv

**Affiliations:** Division of Family Medicine, Centre for Family Medicine, Department of Neurobiology, Care Sciences and Society, Karolinska Institutet, Huddinge, Sweden; Department of Medical Sciences, Cardiovascular Epidemiology, Uppsala University, Uppsala, Sweden; Department of Medical and Health Sciences, Linköping University, Linköping, Sweden; School of Health and Social Studies, Dalarna University, Falun, Sweden

**Keywords:** Type 2 diabetes, TNF, Laplace regression, Incident cardiovascular disease, Mortality, Inflammation

## Abstract

**Aims/hypothesis:**

Soluble tumor necrosis factor receptors 1 and 2 (sTNFR1 and sTNFR2) contribute to experimental diabetic kidney disease, a condition with substantially increased cardiovascular risk when present in patients. Therefore, we aimed to explore the levels of sTNFRs, and their association with prevalent kidney disease, incident cardiovascular disease, and risk of mortality independently of baseline kidney function and microalbuminuria in a cohort of patients with type 2 diabetes. In pre-defined secondary analyses we also investigated whether the sTNFRs predict adverse outcome in the absence of diabetic kidney disease.

**Methods:**

The CARDIPP study, a cohort study of 607 diabetes patients [mean age 61 years, 44 % women, 45 cardiovascular events (fatal/non-fatal myocardial infarction or stroke) and 44 deaths during follow-up (mean 7.6 years)] was used.

**Results:**

Higher sTNFR1 and sTNFR2 were associated with higher odds of prevalent kidney disease [odd ratio (OR) per standard deviation (SD) increase 1.60, 95 % confidence interval (CI) 1.32–1.93, p < 0.001 and OR 1.54, 95 % CI 1.21–1.97, p = 0.001, respectively]. In Cox regression models adjusting for age, sex, glomerular filtration rate and urinary albumin/creatinine ratio, higher sTNFR1 and sTNFR2 predicted incident cardiovascular events [hazard ratio (HR) per SD increase, 1.66, 95 % CI 1.29–2.174, p < 0.001 and HR 1.47, 95 % CI 1.13–1.91, p = 0.004, respectively]. Results were similar in separate models with adjustments for inflammatory markers, HbA1c, or established cardiovascular risk factors, or when participants with diabetic kidney disease at baseline were excluded (p < 0.01 for all). Both sTNFRs were associated with mortality.

**Conclusions/Interpretations:**

Higher circulating sTNFR1 and sTNFR2 are associated with diabetic kidney disease, and predicts incident cardiovascular disease and mortality independently of microalbuminuria and kidney function, even in those without kidney disease. Our findings support the clinical utility of sTNFRs as prognostic markers in type 2 diabetes.

## Background

Diabetes is one of the most important risk factors for cardiovascular disease [1, 2], and the risk of cardiovascular events and complications is substantially higher in those with diabetic kidney disease than in those without [3]. Soluble receptors for tumor necrosis factor (TNF)-α (sTNFRs (sTNFR1 and sTNFR2)), have been suggested to be important factors underpinning the development of diabetic kidney disease in experimental studies [4].

The sTNFRs have earlier been shown to be cross-sectionally associated with lower glomerular filtration rate (GFR) [5], and higher levels of low grade albuminuria in patients with type 2 diabetes [6, 7], as well as ESRD in American Indians with type 2 diabetes [8]. Higher levels of sTNFR2 has also seen on obese than in non-obese patients with type 2 diabetes [9]. Furthermore, in clinical studies in type 1 diabetes, levels of the sTNFRs predict the progression from micro- to macro-albuminuria [10], microalbuminuria and GFR decline [11, 12], and development of CKD stage 3 [13]. Yet, initiation of antidiabetic therapy did not seem to reduce the levels of sTNFR1 or sTNFR2 in in a randomized trial of patients with type 2 diabetes [14].

High levels of sTNFR1 have been associated with increased risk for mortality in various settings such as in patients with rheumatoid arthritis [15], in chronic kidney disease [16], and in the community [17, 18]. Higher levels of sTNFR1 has also been associated with ESRD and mortality in type 2 diabetes [19]. In addition, sTNFR1 and sTNFR2 have been associated with ESRD [20], and as sTNFR1 and sTNFR2 as risk markers for cardiovascular death in type 2 diabetes patients [21]. Moreover, higher levels of sTNFR2 have been shown to be associated with coronary heart disease in women with diabetes [22]. Whether this association is mediated by kidney damage or dysfunction has not been reported.

We hypothesized that sTNFR1 and sTNFR2 are important factors in the development of diabetic kidney disease and cardiovascular disease in patients with type 2 diabetes. Accordingly, we aimed to explore the levels of sTNFRs, and their association with prevalent kidney disease, incident cardiovascular disease, and risk of mortality independently of baseline kidney function and microalbuminuria in a cohort of patients with type 2 diabetes. In pre-defined secondary analyses we also investigated whether the sTNFRs predict adverse outcome in the absence of diabetic kidney disease.

## Methods

Data from the cardiovascular risk factors in patients with diabetes: a Prospective Study in Primary Care (CARDIPP) was analyzed. The study was launched in 2005 and baseline data collection was completed in November 2008. The general aim of CARDIPP was to investigate cardiovascular risk factors in middle-aged patients with type 2 diabetes to facilitate early and individually adjusted risk intervention. Patients aged 55–65 years were consecutively recruited during their usual annual follow-ups at 22 primary healthcare centers in the counties of Östergötland and Jönköping, Sweden, irrespective of their previous blood pressure and CVD status. The centers varied in size and were located in different geographical areas, but all followed the national guidelines for diabetes care. Patients with severe other concomitant diseases such as cognitive impairment and cancer were not included. Yet, we did not exclude patients with severe kidney dysfunction or massive proteinuria.

A questionnaire was used to gain information about lifestyle habits, including alcohol consumption and smoking status, while a standardized medical history provided data on diabetes duration, medications and previous cardiovascular events.

One single spot urine and blood samples for laboratory analyses were taken in the morning following at least 10 h of fasting. Blood samples for the laboratory analyses were collected at the healthcare centers. Clinical data such as body weight, height and waist circumference were also recorded at each primary healthcare center.

Blood pressure was calculated as the average of three sitting measurements taken 1-min apart by specially trained nurses at each primary healthcare center.

Altogether, CARDIPP enrolled 761 participants, out of whom 58 had missing serum samples in the biobank. An additional 71 individuals had suffered a myocardial infarction or stroke prior to the baseline and were excluded from the present analyses. After exclusion of individuals with missing data on estimated GFR (eGFR) or albumin-creatinine ratio (ACR) at the baseline, 607 individuals remained in the present analyses.

The present study was approved by the regional ethics review board based in Linköping, and all participants gave their written informed consent.

### Laboratory analyses

The Swedish standard high-performance liquid chromatography (HPCL) Mono-S method was used to measure HbA1c, although the data were then converted to Diabetes Control and Complications Trial (DCCT) standards (%) and International Federation of Clinical Chemistry and Laboratory Medicine (IFCC) units (mmol/mol). Serum and urine creatinine were measured at the local clinical chemistry laboratory (SWEDAC accredited Lab Med in Östergötland, Sweden) using an IDMS calibrated modified Jaffe method on an Advia 1800 analyzer (Siemens Healthcare Diagnostics). Coefficients of variation (CV) for the creatinine method was 4.3 % at 90 μmol/L and 3.1 % at 380 μmol/L. Urine albumin was analyzed on the same instrument as creatinine using reagents from Siemens Healthcare Diagnostics.

Glomerular filtration rate (eGFR) was estimated using the CKD-EPI formula, and urinary ACR was used as a measure of albuminuria. Kidney dysfunction was defined as having an eGFR <60 mL/min/1.73 m^2^ and microalbuminuria was defined as having ACR <3 (g/mol). Diabetic kidney disease was defined as having kidney dysfunction and/or microalbuminuria at baseline. C-reactive protein (CRP) was measured using a high sensitive latex-enhanced turbidimetric immunoassay (Roche Diagnostics, Mannheim, Germany) with a lower limit of detection of 0.03 mg/L. The coefficient of variation (CV) for the CRP assay was 4.5 %. Plasma levels of IL-6 were measured with an ultrasensitive cytokine bead kit (Invitrogen Co, Carlsbad, CA, USA) according to the manufacturer's instructions and analyzed on a Luminex^®^ 100TM system (Austin, TX, USA). The limit of detection was 0.84 pg mL-1 for IL-6. The intra-assay CV was 5–12 % and the inter-assay CV was 17–20 %.

Blood and urine samples were frozen for later analyses, and the soluble receptors, sTNFR1 and sTNFR2 were analyzed using commercially available ELISA kit (DY225 and DY726, R and D Systems, Minneapolis, MN). The assays had a total CV of approximately 6 %. All laboratory tests were performed blinded without any knowledge of patient outcome.

### Follow-up of outcomes

Incident cardiovascular disease was defined as having a fatal or non-fatal myocardial infarction or stroke diagnosis (International Classification of Diseases, ICD-10 I21 or I63, respectively) recorded in the National In-Hospital Care Register, or any mortality due to cardiovascular causes in the National Cause of Death Register (ICD 10: I00–I99). Total mortality was defined as being registered with a death-date in the National Cause of Death Register. There was no loss of follow-up.

### Statistical analyses

In cross-sectional analyses, medians and their confidence intervals were estimated with Bonett-Price 95 % confidence intervals (CI). Spearman correlation tests were used to study the correlation between sTNFR1 and sTNFR2, as well as between the receptors and the established kidney disease measures eGFR and ACR. Age- and sex adjusted logistic regression was used to calculate the odds ratios (OR) for having prevalent kidney dysfunction, microalbuminuria or kidney disease at baseline.

In longitudinal analyses, Cox regression was used to calculate hazard ratios (HR) with 95 % CI, and Laplace regression was used to calculate the difference in time (in years) until 5 % of the individuals had a cardiovascular event or died during follow up [23]. Bootstrap methods were used to calculate the 95 % CIs in Laplace Regression, with an amendment for Laplace regression to Stata (College Station, Texas, USA), version 11.2. We modelled sTNFR1 and sTNFR2 as per SD increment.

To limit the risk of overfitting, the following two multivariable models were used:

- Model A: adjusted for age, and sex.
- Model B: adjusted for age, sex, eGFR and ACR (primary model).

In secondary models we also added the following adjustments to model A: inflammation [CRP and IL-6 (Model C)], diabetes treatment [HbA1c, and diabetes treatment (diet, oral antidiabetics or insulin) (Model D)], and all factors in model B and established cardiovascular risk factors (smoking, LDL-cholesterol, HDL-cholesterol, systolic blood pressure), cardiovascular medication, antihypertensive medication [ACE-inhibitors/ARBs, calcium channel blockers, beta blockers and diuretics], and lipid lowering treatment (statins)], (Model E).

Multiple imputation were used to account for missing data for co-variates in the Cox regression analyses. As a secondary analysis, we analyzed the risks for incident cardiovascular disease and mortality in individuals without diabetic kidney disease at baseline (n = 467).

We also mirrored the cardiovascular risk with higher levels of sTNFR1 and sTNFR2 using spline-curves.

## Results

Baseline characteristics are shown in Table 1. The mean level of sTNFR1 was 2701 (standard deviation (SD) 854) ng/l, and the mean sTNFR2 was 6383 (SD 2120) ng/l.

**Table 1:** Baseline characteristics

n (%) or median (95 % Bonett-Price confidence interval)	
Number of subjects	607
Men, n. (%)	399 (66 %)
sTNFR1 (ng/l)	2567 (2488–2645)
sTNFR2 (ng/l)	6010 (5818–6202)
Age (years)	61 (60.5–61.5)
Current smoking	108 (18 %)
Glomerular filtration (ml/min/1.73 m^2^)	77 (75–78)
GFR <60 ml/min/1.73 m^2^	58 (10 %)
Albumin-creatinine ratio (g/mol)	0.60 (0.50–0.70)
Microalbuminuria	94 (15 %)
Diabetic kidney disease	140 (23 %)
Systolic blood pressure (mmHg)	137 (135–138)
Diastolic blood pressure (mmHg)	80 (79–81)
HbA1c (IFCC, mmol/mol)	54 (53–56)
LDL-cholesterol (mmol/l)	2.7 (2.6–2.8)
HDL-cholesterol (mmol/l)	1.2 (1.15–1.25)
High sensitivity C-reactive protein (mg/l)	1.60 (1.35–1.85)
Interleukin-6 (ng/l)	2.15 (1.99–2.31)
Statin treatment	327 (53 %)
Antihypertensive treatment	390 (64 %)

### Cross-sectional analyses

The correlation between sTNFR1 and sTNFR2 was R = 0.72 (p < 0.001). Both receptors were also statistically significantly correlated with eGFR; correlation with sTNFR1 was R = −0.21 (p < 0.001), and sTNFR2 R = −0.21 (p < 0.001). Weak correlations with low grade albuminuria were seen; sTNFR1 R = 0.098 (p = 0.015), and sTNFR2 R = 0.052 (p = 0.20).

Logistic regression models for the age and sex adjusted association between SD increments in sTNFR1 and sTNFR2, and kidney dysfunction, microalbuminuria and diabetic kidney disease are shown in Table 2. Both receptors were significantly associated with all three kidney phenotypes.

**Table 2:** Logistic regression models for the age and sex adjusted association between sTNFR1 and sTNFR2, and kidney dysfunction (GFR <60 ml/min/1.73 m^2^, n = 58), microalbuminuria (n = 94), or diabetic kidney disease (either low kidney dysfunction, microalbuminuria, or both n = 140)

	Odds ratio (per SD)	95 % CI	p value
sTNFR1			
GFR <60 ml/min/1.73 m^2^	1.87	1.47–2.38	<0.001
Microalbuminuria	1.32	1.07–1.63	0.009
Diabetic kidney disease	1.60	1.32–1.93	<0.001
sTNFR2			
GFR <60 ml/min/1.73 m^2^	1.54	1.21–1.97	0.001
Microalbuminuria	1.36	1.10–1.67	0.004
Diabetic kidney disease	1.43	1.19–1.71	<0.001

### Longitudinal analyses

During follow-up (mean 7.4 years) a total of 45 participants had an incident cardiovascular event and 44 participants died (incidence rate 1.00 (95 % CI 0.74–1.34) and 0.94 (95 % CI 0.71–1.28) per 100 person years of follow-up, respectively (16 died of cardiovascular causes, 16 died of cancer, 3 died of diabetes, and 9 died of other causes). In the subsample without diabetic kidney disease at baseline (n = 467), 27 participants had an incident cardiovascular event and (mean 7.6 years) 32 participants died (incidence rate 0.77 (95 % CI 0.53–1.13) and 0.90 (95 % CI 0.64–1.27) per 100 person years of follow-up, respectively).

**Fig. 1:**
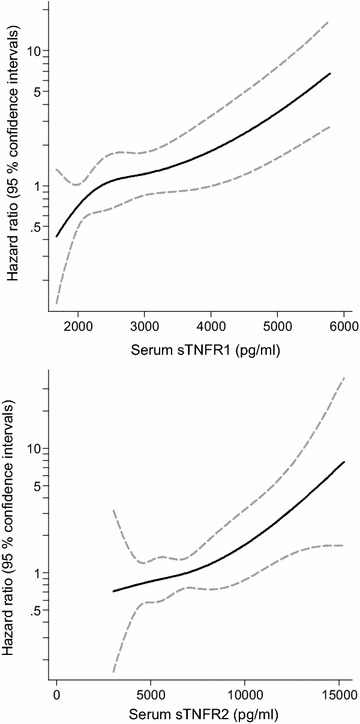
Regression spline curves with 95 % confidence intervals of the unadjusted association between higher levels of sTNFR1 and sTNFR2 with the risk of cardiovascular disease

Cox regression models of the risk of incident cardiovascular events associated with increments in sTNFR1 and sTNFR2 are shown in Table 3 in all individuals as well as in those without kidney disease at baseline. Higher risk estimates were seen with higher levels of both receptors, in primary multivariable models (Model A–D). The spline curves (Fig. 1) suggest no deviation from linearity in the association between the sTNFRs and cardiovascular events. In participants without kidney disease at baseline, both sTNFR1 and sTNFR2 predicted cardiovascular events in all multivariable models (Table 3).

**Table 3:** Multivariable Cox regression models showing the association between sTNFR1 and sTNFR2, with major cardiovascular events in all individuals and in those without kidney disease

Biomarker	Model	Hazard ratio	95 % confidence intervals	p value
sTNFR1 all	A	1.68	(1.33–2.13)	<0.001
	B	1.66	(1.29–2.14)	<0.001
	C	1.61	(1.26–2.06)	<0.001
	D	1.65	(1.29–2.11)	<0.001
	E	1.79	(1.39–2.14)	<0.001
Without kidney disease	A	1.88	(1.42–2.25)	<0.001
	B	1.94	(1.47–2.56)	<0.001
	C	1.77	(1.32–2.39)	<0.001
	D	1.85	(1.39–2.47)	<0.001
	E	2.06	(1.51–2.82)	<0.001
sTNFR2 all	A	1.51	(1.17–1.93)	0.001
	B	1.47	(1.13–1.91)	0.004
	C	1.44	(1.10–1.88)	0.008
	D	1.46	(1.13–1.89)	0.004
	E	1.48	(1.14–1.93)	0.003
Without kidney disease	A	1.67	(1.25–2.25)	0.001
	B	1.72	(1.29–2.28)	<0.001
	C	1.56	(1.13–2.17)	0.008
	D	1.70	(1.26–2.30)	0.001
	E	1.74	(1.26–2.40)	0.001

Model A: adjusted for age, and sex; Model B: adjusted for age, sex, eGFR and ACR; Model C: adjusted for age, sex, CRP IL-6; Model D: adjusted for age, sex, HbA1c, and diabetes treatment (diet, oral antidiabetics or insulin); Model E (secondary analysis): established cardiovascular risk factors (smoking, LDL-cholesterol, HDL-cholesterol, systolic blood pressure), cardiovascular medication, antihypertensive medication (ACE-inhibitors/ARBs, calcium channel blockers, beta blockers and diuretics), and lipid lowering treatment (statins), to model B

In Laplace regression models adjusted for age, sex, GFR and low grade albuminuria (Model B) a standard deviation higher level of sTNFR1 and sTNFR2 was associated with a shorter estimated time until 5 % of the participants suffered an incident cardiovascular disease event: (1.65 years, (95 % CI 3.02–0.28 years), p = 0.018 and 1.38 years (95 % CI 2.14–0.62 years), p < 0.001, respectively.

The Cox regression risk estimates for the association between sTNFR1 and sTNFR2 and total mortality associated with standard deviation increments in sTNFR1 and sTNFR2 are shown in Table 4 in all individuals as well as in those without kidney disease at baseline. Higher risks were seen with higher levels of both receptors, but the risk estimates were slightly higher for sTNFR2 than for sTNFR1. The elevated risks were statistically significant in all models tested.

**Table 4:** Multivariable Cox regression models showing the association between sTNFR1 and sTNFR2, with mortality in all individuals and in those without kidney disease

Biomarker	Model	Hazard ratio	95 % confidence intervals	p value
sTNFR1 all	A	1.47	(1.19–1.83)	<0.001
	B	1.50	(1.21–1.86)	<0.001
	C	1.40	(1.11–1.76)	0.004
	D	1.49	(1.19–1.86)	<0.001
	E	1.45	(1.14–1.85)	0.003
Without kidney disease	A	1.65	(1.32–2.05)	<0.001
	B	1.67	(1.34–2.09)	<0.001
	C	1.58	(1.25–2.00)	<0.001
	D	1.71	(1.35–2.17)	<0.001
	E	1.70	(1.28–2.25)	<0.001
sTNFR2	A	1.50	(1.18–1.91)	0.001
	B	1.52	(1.19–1.94)	0.001
	C	1.41	(1.08–1.83)	0.010
	D	1.51	(1.18–1.93)	0.001
	E	1.58	(1.20–2.09)	0.001
Without kidney disease	A	1.65	(1.28–2.13)	<0.001
	B	1.69	(1.31–2.18)	<0.001
	C	1.54	(1.16–2.05)	0.003
	D	1.66	(1.28–2.15)	<0.001
	E	1.95	(1.40–2.70)	<0.001

Model A: adjusted for age, and sex; Model B: adjusted for age, sex, eGFR and ACR; Model C: adjusted for age, sex, CRP IL-6; Model D: adjusted for age, sex, HbA1c, and diabetes treatment (diet, oral antidiabetics or insulin); E (secondary analysis): established cardiovascular risk factors (smoking, LDL-cholesterol, HDL-cholesterol, systolic blood pressure), cardiovascular medication, antihypertensive medication (ACE-inhibitors/ARBs, calcium channel blockers, beta blockers and diuretics), and lipid lowering treatment (statins), to model B

In Laplace regression models adjusted for age, sex, smoking, eGFR and low grade albuminuria (Model B) a standard deviation higher level of sTNFR1 was associated with a shorter estimated time until 5 % of the participants died: 1.85 years, (95 % CI 2.83–0.88 years), p < 0.001. Using Model B, but sTNFR2 instead of sTNFR1, one standard deviation higher levels of sTNFR2 was associated with 2.35 years (95 % CI 3.53–1.17 years), p < 0.001, until the first 5 % died.

There were no missing in model A and model B, but the imputed missing data were useful in other models, where complete data on all variables was available for: model C n = 586, model D n = 604, and model E n = 559.

## Discussion

In the present study, higher levels of both sTNFR1 and sTNFR2 were associated with prevalent diabetic kidney disease. Yet, both sTNFRs robustly predicted incident cardiovascular events independently of age, sex, and established kidney disease measures. The results were also significant in separate models adjusted for cardiovascular risk factors, cardiovascular medication, inflammatory markers, and HbA1c. Interestingly, both receptors were associated with an increased risk also in those without kidney disease at baseline indicating that sTNFR1 and sTNFR2 portray prognostic information prior to the development of overt diabetic nephropathy. Robust associations with total mortality were also seen for both sTNFR1 and sTNFR2.

### Comparisons with other studies

In line with previous studies in type 2 diabetes patients, high levels of sTNFR1 and sTNFR2 were associated with worsened kidney function and higher urinary albumin/creatinine ratio [5–7, 24].

As summarized in the introduction, some evidence of adverse outcomes in type 2 diabetes has been published. Higher levels of sTNFR1 was associated with increased mortality risk in patients with type 2 diabetes and kidney disease [21]. Another trial showed that higher sTNFR2 predicted coronary heart disease in females with type 2 diabetes [22], yet, the authors of that study did not investigate whether the results were significant after adjustments for kidney function or albuminuria, which is the novelty of the present study. We also showed that the associations were present in individuals without prevalent kidney disease.

### Potential mechanistic pathways for our observations

The association between sTNFR1 and sTNFR2 and incident cardiovascular disease could be explained by their atherosclerotic effects, or by being markers of systemic inflammation [22, 25]. Inflammation plays a vital part in the formation of the atherosclerotic lesion, as well as in plaque rupture; the final trigger of most ischemic cardiovascular events [26]. However, the associations with incident cardiovascular disease were independent of established cardiovascular risk factors, including hsCRP and IL6, showing that levels of sTNFRs may provide additional information in cardiovascular prediction.

Hyperglycemia has been shown to affect the levels of oxidative stress [27, 28]. Oxidative stress increase the overall TNF-α system activity [27], especially sTNFR2 [29]. Hyperglycemia may also cause arterial stiffness. Pulse wave velocity is a common surrogate for arterial stiffness and has been shown to be highly correlated with the levels of sTNFR1 and sTNFR2 [30].

Higher levels of sTNFR1 and sTNFR2 have been shown to be associated with development of ESRD in patients with type-1 diabetes without proteinuria [12], as well as risk for early renal decline in individuals with type-1 diabetes with normal renal function (and no proteinuria) [13]. A more rapid decline in kidney function and development of diabetes nephropathy may thus explain the associations between sTNFR1 and sTNFR2 with cardiovascular events and with mortality that we observed, as rapid progression of nephropathy has been associated with poor prognoses in individuals with diabetes [3, 4]. It should also be noted that the sTNFRs predicted adverse outcome independently of HbA1c which suggest that poor glucose control is not a main driver of the associations.

The association between sTNFR1 and sTNFR2 and mortality may have several explanations, as TNF-α and its soluble receptors are important in several elementary pathological processes not only in cardiovascular disease but also cancer [31, 32]. In fact, higher levels of sTNFR1 and sTNFR2 are likely to be associated with lower cancer survival [33–35]. The joint action of interferon-gamma and TNF-α may lead to cell cycle arrest in most cancers, however, this phenomenon requires the tumor to express sTNFR1 [36]. The role of sTNFR1 and sTNFR2 as risk factors of cause-specific mortality in individuals with diabetes requires further studies in larger study samples.

### Clinical implications

Given the ongoing global diabetes epidemic it will be of increasing importance in the future to discriminate between those patients with an increased risk of a rapid progression to complications and death, from those with a slow and more benign progression. Our findings put forward that high levels of sTNFRs are potentially useful markers of poor prognosis in type 2 diabetes, in addition to their potential use as markers of kidney function decline, progression of chronic kidney disease and nephropathy [10–13, 37]. The TNF superfamily, with TNF-alpha, sTNFR1 and sTNFR2, TNF-like weak inducer of apoptosis (sTWEAK) [38], tumor necrosis factor receptor superfamily member 6 [39], Tumor necrosis factor ligand superfamily member 14 (TNFSF14) [40], and other member proteins are a part of a complex interplay; and that different ways to clinically target these pathways are possible. The biguanide buformin, which is closely related to the most commonly used antidiabetic drug metformin was not associated with any changes in the levels of sTNFR1, in a study of obese patients with type 2 diabetes where buformin was compared to lifestyle changes [41]. However, pioglitazone was associated with fewer cardiovascular events in the PROspective pioglitAzone Clinical Trial In macroVascular Events (PROactive study) [42], and mechanistic studies in humans have shown that pioglitazone can modulate the increased activity of TNF-α converting enzyme, in patients with type 2 diabetes and obesity [43]. Moreover, aspirin has been shown to reduce TNF-α in patients with type 2 diabetes [44]. Anti-TNF therapy has been studied in patients with cancer [33], cardiovascular disease [45], as well as kidney disease [46, 47], whether also anti-TNF therapy can be beneficial in Type 2 diabetes remains, as far as we know, to be studied.

### Strengths and limitations

Strengths of our investigation include the prospective longitudinal study design, the detailed characterization of study participants, and relatively large sample of diabetes patients with no loss to follow-up. Limitations include the observational design, and extrapolation to other ethnic groups. There were also a fairly low rate of incident cardiovascular disease and mortality, limiting the possibility to adjust for many factors in the same models, which is why we classified the model adjusted for all the cardiovascular risk factors as a secondary model. The limited number of events also limited us from trying to find a reliable cut-off for higher risk with sTNFR1 and sTNFR2, and from studying ESRD as an outcome. Furthermore, regarding the data on microalbuminuria, our study was limited by having only a single spot urine sample.

## Conclusions

Our data confirm and extend previous studies suggesting that sTNFR1 and sTNFR2 are promising biomarkers for diabetic kidney disease and cardiovascular risk independently of microalbuminuria and baseline kidney function, i.e. also in the early stages of type 2 diabetes, as well as in early stages of diabetic nephropathy. Further studies are warranted to evaluate the future clinical role and use of sTNFR1 and sTNFR2 as markers to identify diabetes patients with high risk for micro- and macro-vascular complications.

## References

[1] Greenland P, Knoll MD, Stamler J, Neaton JD, Dyer AR, Garside DB, Wilson PW (2003). Major risk factors as antecedents of fatal and nonfatal coronary heart disease events. JAMA.

[2] Khot UN, Khot MB, Bajzer CT, Sapp SK, Ohman EM, Brener SJ, Ellis SG, Lincoff AM, Topol EJ (2003). Prevalence of conventional risk factors in patients with coronary heart disease. JAMA.

[3] Palsson R, Patel UD (2014). Cardiovascular complications of diabetic kidney disease. Adv Chronic Kidney Dis.

[4] Hasegawa G, Nakano K, Sawada M, Uno K, Shibayama Y, Ienaga K, Kondo M (1991). Possible role of tumor necrosis factor and interleukin-1 in the development of diabetic nephropathy. Kidney Int.

[5] Izumi Y, Yabe D, Taniguchi A, Fukushima M, Nakai Y, Hosokawa M, Okumura T, Nin K, Matsumoto K, Nishimura F (2013). Circulating TNF receptor 2 is associated with the development of chronic kidney disease in non-obese Japanese patients with type 2 diabetes. Diabetes Res Clin Pract.

[6] Vendrell J, Broch M, Fernandez-Real JM, Gutierrez C, Simon I, Megia A, Gallart L, Ricart W, Richart C (2005). Tumour necrosis factor receptors (TNFRs) in Type 2 diabetes. Analysis of soluble plasma fractions and genetic variations of TNFR2 gene in a case-control study. Diabet Med.

[7] Kawasaki Y, Taniguchi A, Fukushima M, Nakai Y, Kuroe A, Ohya M, Nagasaka S, Yamada Y, Inagaki N, Seino Y (2005). Soluble TNF receptors and albuminuria in non-obese Japanese type 2 diabetic patients. Horm Metab Res.

[8] Pavkov ME, Nelson RG, Knowler WC, Cheng Y, Krolewski AS, Niewczas MA (2015). Elevation of circulating TNF receptors 1 and 2 increases the risk of end-stage renal disease in American Indians with type 2 diabetes. Kidney Int.

[9] Chearskul S, Sriwijitkamol A, Kooptiwut S, Ornreabroi S, Churintaraphan M, Samprasert N (2015). Cardiometabolic risk in Thai adults with type 2 diabetes mellitus: obese versus non-obese. J Med Assoc Thai.

[10] Lopes-Virella MF, Baker NL, Hunt KJ, Cleary PA, Klein R, Virella G (2013). Baseline markers of inflammation are associated with progression to macroalbuminuria in type 1 diabetic subjects. Diabetes Care.

[11] Krolewski AS, Niewczas MA, Skupien J, Gohda T, Smiles A, Eckfeldt JH, Doria A, Warram JH. Early progressive renal decline precedes the onset of microalbuminuria and its progression to macroalbuminuria. Diabetes care. 2013. 10.2337/dc13-0985 PMC3867993 23939543

[12] Niewczas MA, Ficociello LH, Johnson AC, Walker W, Rosolowsky ET, Roshan B, Warram JH, Krolewski AS (2009). Serum concentrations of markers of TNFalpha and Fas-mediated pathways and renal function in nonproteinuric patients with type 1 diabetes. Clin J Am Soc Nephrol.

[13] Gohda T, Niewczas MA, Ficociello LH, Walker WH, Skupien J, Rosetti F, Cullere X, Johnson AC, Crabtree G, Smiles AM (2012). Circulating TNF receptors 1 and 2 predict stage 3 CKD in type 1 diabetes. J Am Soc Nephrol.

[14] Pradhan AD, Everett BM, Cook NR, Rifai N, Ridker PM (2009). Effects of initiating insulin and metformin on glycemic control and inflammatory biomarkers among patients with type 2 diabetes: the LANCET randomized trial. JAMA.

[15] Mattey DL, Glossop JR, Nixon NB, Dawes PT (2007). Circulating levels of tumor necrosis factor receptors are highly predictive of mortality in patients with rheumatoid arthritis. Arthritis Rheum.

[16] Neirynck N, Glorieux G, Schepers E, Verbeke F, Vanholder R (2015). Soluble tumor necrosis factor receptor 1 and 2 predict outcomes in advanced chronic kidney disease: a prospective cohort study. PLoS One.

[17] Carlsson AC, Juhlin CC, Larsson TE, Larsson A, Ingelsson E, Sundstrom J, Lind L, Arnlov J (2014). Soluble tumor necrosis factor receptor 1 (sTNFR1) is associated with increased total mortality due to cancer and cardiovascular causes - findings from two community based cohorts of elderly. Atherosclerosis.

[18] Luna JM, Moon Y, Liu K, Spitalnik S, Paik M, Sacco R, Elkind MS (2013). Tumour necrosis factor receptor 1 and mortality in a multi-ethnic cohort: the Northern Manhattan Study. Age Ageing.

[19] Lee JE, Gohda T, Walker WH, Skupien J, Smiles AM, Holak RR, Jeong J, McDonnell KP, Krolewski AS, Niewczas MA (2013). Risk of ESRD and all cause mortality in type 2 diabetes according to circulating levels of FGF-23 and TNFR1. PLoS One.

[20] Niewczas MA, Gohda T, Skupien J, Smiles AM, Walker WH, Rosetti F, Cullere X, Eckfeldt JH, Doria A, Mayadas TN (2012). Circulating TNF receptors 1 and 2 predict ESRD in type 2 diabetes. J Am Soc Nephrol.

[21] Saulnier PJ, Gand E, Ragot S, Ducrocq G, Halimi JM, Hulin-Delmotte C, Llaty P, Montaigne D, Rigalleau V, Roussel R (2014). Association of serum concentration of TNFR1 with all-cause mortality in patients with type 2 diabetes and chronic kidney disease: follow-up of the SURDIAGENE cohort. Diabetes Care.

[22] Shai I, Schulze MB, Manson JE, Rexrode KM, Stampfer MJ, Mantzoros C, Hu FB (2005). A prospective study of soluble tumor necrosis factor-alpha receptor II (sTNF-RII) and risk of coronary heart disease among women with type 2 diabetes. Diabetes Care.

[23] Bottai M, Zhang J (2010). Laplace regression with censored data. Biom J.

[24] Miyazawa I, Araki S, Obata T, Yoshizaki T, Morino K, Kadota A, Ugi S, Kawai H, Uzu T, Nishio Y (2011). Association between serum soluble TNFalpha receptors and renal dysfunction in type 2 diabetic patients without proteinuria. Diabetes Res Clin Pract.

[25] Safranow K, Dziedziejko V, Rzeuski R, Czyzycka E, Wojtarowicz A, Binczak-Kuleta A, Jakubowska K, Olszewska M, Ciechanowicz A, Kornacewicz-Jach Z (2009). Plasma concentrations of TNF-alpha and its soluble receptors sTNFR1 and sTNFR2 in patients with coronary artery disease. Tissue Antigens.

[26] Libby P (2012). Inflammation in atherosclerosis. Arterioscler Thromb Vasc Biol.

[27] Esposito K, Nappo F, Marfella R, Giugliano G, Giugliano F, Ciotola M, Quagliaro L, Ceriello A, Giugliano D (2002). Inflammatory cytokine concentrations are acutely increased by hyperglycemia in humans: role of oxidative stress. Circulation.

[28] Evans JL, Goldfine ID, Maddux BA, Grodsky GM (2002). Oxidative stress and stress-activated signaling pathways: a unifying hypothesis of type 2 diabetes. Endocr Rev.

[29] Fernandez-Real JM, Broch M, Ricart W, Casamitjana R, Gutierrez C, Vendrell J, Richart C (1998). Plasma levels of the soluble fraction of tumor necrosis factor receptor 2 and insulin resistance. Diabetes.

[30] Ohgushi M, Taniguchi A, Fukushima M, Nakai Y, Kuroe A, Ohya M, Nagasaka S, Taki Y, Yoshii S, Matsumoto K (2007). Soluble tumor necrosis factor receptor 2 is independently associated with pulse wave velocity in nonobese Japanese patients with type 2 diabetes mellitus. Metabolism.

[31] Cabal-Hierro L, Lazo PS (2012). Signal transduction by tumor necrosis factor receptors. Cell Signal.

[32] Pass HI, Mew D, Pass HA, Temeck BK (1995). The macrophage, TNF, and other cytokines. Chest Surg Clin N Am.

[33] Askling J, Baecklund E, Granath F, Geborek P, Fored M, Backlin C, Bertilsson L, Coster L, Jacobsson LT, Lindblad S (2009). Anti-tumour necrosis factor therapy in rheumatoid arthritis and risk of malignant lymphomas: relative risks and time trends in the Swedish Biologics Register. Ann Rheum Dis.

[34] Dobrzycka B, Terlikowski SJ, Kowalczuk O, Kinalski M (2009). Circulating levels of TNF-alpha and its soluble receptors in the plasma of patients with epithelial ovarian cancer. Eur Cytokine Netw.

[35] Dossus L, Becker S, Rinaldi S, Lukanova A, Tjonneland A, Olsen A, Overvad K, Chabbert-Buffet N, Boutron-Ruault MC, Clavel-Chapelon F (2011). Tumor necrosis factor (TNF)-alpha, soluble TNF receptors and endometrial cancer risk: the EPIC study. Int J Cancer.

[36] Braumuller H, Wieder T, Brenner E, Assmann S, Hahn M, Alkhaled M, Schilbach K, Essmann F, Kneilling M, Griessinger C (2013). T-helper-1-cell cytokines drive cancer into senescence. Nature.

[37] Carlsson AC, Larsson TE, Helmersson-Karlqvist J, Larsson A, Lind L, Arnlov J (2014). Soluble TNF receptors and kidney dysfunction in the elderly. J Am Soc Nephrol.

[38] Diaz-Lopez A, Bullo M, Chacon MR, Estruch R, Vendrell J, Diez-Espino J, Fito M, Corella D, Salas-Salvado J (2014). Reduced circulating sTWEAK levels are associated with metabolic syndrome in elderly individuals at high cardiovascular risk. Cardiovasc Diabetol.

[39] Lind L, Siegbahn A, Lindahl B, Stenemo M, Sundstrom J, Arnlov J (2015). Discovery of new risk markers for ischemic stroke using a novel targeted proteomics chip. Stroke.

[40] Lind L, Arnlov J, Lindahl B, Siegbahn A, Sundstrom J, Ingelsson E (2015). Use of a proximity extension assay proteomics chip to discover new biomarkers for human atherosclerosis. Atherosclerosis.

[41] Ohara S, Komatsu R, Matsuyama T (2004). Short-term effect of buformin, a biguanide, on insulin sensitivity, soluble fraction of tumor necrosis factor receptor and serum lipids in overweight patients with type 2 diabetes mellitus. Diabetes Res Clin Pract.

[42] Dormandy JA, Charbonnel B, Eckland DJ, Erdmann E, Massi-Benedetti M, Moules IK, Skene AM, Tan MH, Lefebvre PJ, Murray GD (2005). Secondary prevention of macrovascular events in patients with type 2 diabetes in the PROactive Study (PROspective pioglitAzone Clinical Trial In macroVascular Events): a randomised controlled trial. Lancet.

[43] Monroy A, Kamath S, Chavez AO, Centonze VE, Veerasamy M, Barrentine A, Wewer JJ, Coletta DK, Jenkinson C, Jhingan RM (2009). Impaired regulation of the TNF-alpha converting enzyme/tissue inhibitor of metalloproteinase 3 proteolytic system in skeletal muscle of obese type 2 diabetic patients: a new mechanism of insulin resistance in humans. Diabetologia.

[44] Derosa G, Mugellini A, Pesce RM, D’Angelo A, Maffioli P (2015). A study about the relevance of adding acetylsalicylic acid in primary prevention in subjects with type 2 diabetes mellitus: effects on some new emerging biomarkers of cardiovascular risk. Cardiovasc Diabetol.

[45] Bozkurt B, Torre-Amione G, Warren MS, Whitmore J, Soran OZ, Feldman AM, Mann DL (2001). Results of targeted anti-tumor necrosis factor therapy with etanercept (ENBREL) in patients with advanced heart failure. Circulation.

[46] Elmarakby AA, Quigley JE, Pollock DM, Imig JD (2006). Tumor necrosis factor alpha blockade increases renal Cyp2c23 expression and slows the progression of renal damage in salt-sensitive hypertension. Hypertension.

[47] Muller DN, Shagdarsuren E, Park JK, Dechend R, Mervaala E, Hampich F, Fiebeler A, Ju X, Finckenberg P, Theuer J (2002). Immunosuppressive treatment protects against angiotensin II-induced renal damage. Am J Pathol.

